# Association between adolescent pregnancy and adverse birth outcomes, a multicenter cross sectional Japanese study

**DOI:** 10.1038/s41598-019-38999-5

**Published:** 2019-02-20

**Authors:** Kohei Ogawa, Sachio Matsushima, Kevin Y. Urayama, Norihiko Kikuchi, Noriyuki Nakamura, Shinji Tanigaki, Haruhiko Sago, Shoji Satoh, Shigeru Saito, Naho Morisaki

**Affiliations:** 1 Center for Maternal-Fetal, Neonatal and Reproductive Medicine, National Center for Child Health and Development, 2-10-1 Okura, Setagaya-ku, Tokyo, 157-8535 Japan; 2 Collaborative Departments of Advanced Pediatric Medicine, Graduate School of Medicine, Tohoku University, 1-1 Seiryo-cho, Aoba-ku, Sendai-shi, Miyagi 980-8575 Japan; 3 Department of Social Medicine, National Center for Child Health and Development, 2-10-1 Okura, Setagaya-ku, Tokyo, 157-8535 Japan; 4 Graduate School of Public Health, St Luke’s International University, 9-1 Akashi-cho, Chuo-ku, Tokyo, 104-8560 Japan; 5 Perinatal Center, Oita Prefectural Hospital, 476 Bunyo, Oita-shi, Oita, 870-8511 Japan; 6 Department of Obstetrics & Gynecology, University of Toyama, School of Medicine, 2630, Sugitani, Toyama-shi, Toyama, 930-0194 Japan

## Abstract

We aimed to clarify how maternal physical characteristics explains the association between adolescent pregnancy and adverse birth outcomes, focusing on their height. We used a national multicenter-based delivery registry among 30,831 women under age 25 years with a singleton pregnancy between 2005 and 2011. Adolescent pregnancy was defined as younger than 20 years of age, and categorized into “junior adolescent” (aged ≤15 years) and “senior adolescent” (aged 16–19 years). We used multivariate Poisson regression and mediation analysis to assess the extent to which maternal height explained the association between adolescent pregnancy and risk of adverse birth outcomes. Risks for preterm birth [(adjusted risk ratio (aRR) 1.17, 95% confidence interval (95% CI), 1.08–1.27], low birthweight (aRR 1.08, 95% CI, 1.01–1.15), and low Apgar score (aRR 1.41 95%CI, 1.15–1.73) were significantly higher among adolescent women compared to women of 20–24 years of age. The mediation effect of maternal height on these outcomes were moderate for low birthweight (45.5%) and preterm birth (10.5%), and smaller for low Apgar score (6.6%). In all analyses, we did not detect significant differences between junior adolescent and senior adolescent. Adolescent women have higher risk of adverse birth outcomes. This association is partially mediated by shorter maternal height.

## Introduction

Adolescent pregnancy has been a target for prevention^1^ in many countries such as the United States^2^, and Japan^3^, as well as low and middle income countries^4,5^. One main reason for this large interest is that adolescent pregnancy, with its many associated social problems such as single parenthood, welfare dependency, maternal low educational attainment, and maternal disability pension, has been known to increase risk of subsequent child abuse, neglect, suicide and double suicide^6–8^. Furthermore, studies show that offspring of adolescent pregnancies are more likely to become teenage parents as well^9^, thus causing a generational chain of people with social and economic risk.

In addition to these potential social and economic repercussions, several studies in both low and higher income countries have shown that adolescent pregnancy is associated with an increased risk of adverse birth outcomes, including preterm birth, low birthweight, neonatal asphyxia and perinatal death^10–15^. However, it is not clear based on current evidence as to what may be explaining this association. Together with possible lifestyle behaviors associated with both adolescent pregnancy^16^ and adverse birth outcomes^17^, it is plausible that physical immaturity in adolescents can be contributing to the association. Studies focusing on gynecologic age, or uterine immaturity, have supported this hypothesis^18^.

As female height continues to increase until 18 to 19 years of age, shorter maternal height would reflect such physical immaturity in adolescents. Shorter height is also known to increase risk of adverse birth outcomes such as preterm delivery, small for gestational age (SGA), and preeclampsia among mature adults^19–22^. However, to our knowledge, none of the previous studies which studied the increase in risk of adverse outcomes in adolescent pregnancies have considered the role of maternal height^11,13,15^.

Using a Japanese national multicenter-based delivery registry, we investigated birth outcomes in Japanese adolescent women, and estimated the extent to which shorter maternal height, a proxy for the immaturity of the maternal body, may mediate this association.

## Methods

### Study population

We used data from the Japan Society of Obstetrics and Gynecology Perinatal Database (JSOG-DB), which has been described elsewhere in detail^22^. Briefly, this database is an ongoing registry which is currently based on 149 Japanese tertiary hospitals and covers over a hundred thousand annual births. In each hospital, maternal demographics, pregnancy complications, and birth outcomes were transcribed from medical charts using a standardized format.

The registry included 54,047 women under 25 years old who gave birth to a singleton with no congenital anomaly between April 2005 and December 2011. Exclusions included women with missing outcome variables (n = 331) or unreliable data on pre-pregnancy weight (<20 kg considered unreliable), height (>4 SD or <−4SD of the population), and birthweight for gestational age^23^ (n = 415). In a large proportion of women, data on maternal height (n = 17,449), maternal body mass index (BMI) (n = 19,387), and gestational weight gain (n = 20,706) were missing; thus, we conducted our main analysis on 30,831 women with complete covariate data. In addition, we conducted analyses on the total 53,301 women using imputed values for the missing data in order to evaluate potential for selection bias and external validity of results. Multiple imputation by chained equations was used to create 100 sets of imputed datasets. For analyses of severe laceration as an outcome, the subjects were restricted to 24,631 women who had never received cesarean-section.

### Variables of interest

Maternal age was our exposure of interest. As our database did not have age at conception, we used age at delivery. We broadly categorized women into young adult (20–24 years of age as a reference group) and adolescent (≤19 years). We further divided adolescent into two sub-categories (junior adolescent as ≤15 years of age and senior adolescent as 16–19 years of age) because previous studies have reported junior adolescents may have even higher risk of adverse outcomes^24,25^.

Primary outcomes of interest were maternal and neonatal complications at birth, including preterm birth, very preterm birth, extremely preterm birth, SGA, intrauterine fetal death (IUFD), cesarean section, unplanned cesarean section, pre-eclampsia, severe preeclampsia, severe vaginal laceration at birth, and neonatal Apgar score at 5 minutes. We defined SGA as birthweight below 10^th^ percentile for the given gestational age using the Japanese birthweight ref.^26^, preterm birth as less than 37 completed weeks of gestation, very preterm birth as less than 32 completed weeks of gestation, and extremely preterm birth as less than 28 completed weeks of gestation^27^. Preeclampsia and severe preeclampsia were diagnosed clinically by obstetricians at each hospital according to national guideline as systolic/diastolic blood pressure over 140/90 mmHg and 160/110 mmHg, respectively, that emerges after 20 weeks gestation with significant proteinuria (≥300 mg/day)^28^. We also defined severe laceration at birth as three or four degree laceration, and low Apgar score at five minutes as below 7.

We considered maternal height, as a mediator of interest. The following factors were also included in the multivariate models: parity, year of delivery, maternal smoking status, pre-existing hypertension, pre-existing diabetes or gestational diabetes, pre-pregnancy BMI, and gestational weight gain during pregnancy^15^. For smoking, a separate category was created for those with missing data.

### Statistical analysis

We compared baseline demographics among the three categories of maternal age using the chi-squared test for categorical variables and one-way ANOVA test for continuous variables.; we also compared the characteristics between 30,831 women with complete covariate data and 22,470 with missing data, using the chi-squared test for categorical variables and student t test for continuous variables. Poisson regression was used to estimate the effect of adolescent pregnancy on the risk of adverse birth outcomes, as well as changes in the effect after sequentially adjusting for demographics and considering maternal height as a mediator. For each outcome, we pursued two separate multivariate models; (1) including adolescent pregnancy, parity, year of delivery, maternal smoking, pre-pregnancy BMI and gestational weight gain (model 1), and (2) including all variables in Model 1 and maternal height (model 2). To confirm the generalizability of our results, we conducted sensitivity analysis on the whole sample (n = 53,301) with missing values of the following variables imputed through multiple imputation with 100 sets of imputations: maternal height (n = 17,449), maternal BMI (n = 19,387), maternal gestational weight gain during pregnancy (n = 20,706) and smoking (n = 15,557). For all Poisson models, we conducted analyses comparing all adolescents with non-adolescents, as well as comparing junior adolescents and senior adolescents with non-adolescents. All results were based on robust variance estimates^29^.

Next, for the subset of adverse outcomes that were associated with adolescent pregnancy (preterm birth, very preterm birth, extremely preterm birth, low birthweight, and low Apgar score), we conducted mediation analysis to estimate the mediated effect of adolescent pregnancy through maternal height. Mediation analysis hypothesizes that the total effect of maternal age (X) on an adverse outcome (Y) consists of a direct effect and an indirect effect (i.e., is mediated by height (M)), and calculates the proportion of the total effect that is indirect (i.e. mediated)^30^. Theoretically this calculation is based on three models, (1) one estimating the total effect (c) of X on Y, (2) one estimating the indirect effect (c’) of X on Y with M included as a covariate, and (3) one estimating the effect of X on M^30^ from which the mediating effect (of X on Y through M) is calculated as a fraction of the total effect (of X on Y). We used an improved analytical method that takes into account possible exposure-mediator interactions as proposed by VanderWeele^31^ rather than crudely calculating the mediating effect by dividing c’ by c as proposed by Baron and Kenny^30^.

All statistical analyses were conducted using the statistical software package Stata SE 14 (STATA Corp, College Station, TX). Statistical significance was set under 0.05, and all statistical tests were two tailed. The protocol for this study was approved by the Institutional Review Board of the National Center for Child Health and Development on Apr. 18, 2017 (No 1448).

## Results

Women in younger categories of age were significantly shorter, thinner, and more likely to gain weight during pregnancy (Table 1). Of 30,831 women, 5,821 (18.9%) experienced low birthweight, and 4,050 (13.1%) experienced preterm birth, including 1,016 very preterm births and 378 extremely preterm births. As for mode of delivery, 6,200 women (20.1%) underwent cesarean section, including 3,690 (12.0%) unplanned surgeries.

**Table 1:** Maternal and infant characteristics by maternal age category among 30,831. Japanese women under 25 years of age.

Mean (SD) or n (%)	Maternal age			p^a^
	Junior adolescent (n = 122)	Senior adolescent (n = 3,863)	Young adult (n = 26,846)	
**Maternal characteristics**				
Maternal age at birth (years)	14.7 (0.6)	18.2 (1.0)	22.5 (1.3)	<0.001
Pre-pregnancy weight (kg)	48.6 (7.4)	50.5 (8.2)	51.5 (9.0)	<0.001
Maternal height (cm)	155.5 (5.6)	156.5 (5.4)	157.4 (5.5)	<0.001
Pre-pregnancy body mass index (kg/m^2^)	20.1 (2.5)	20.6 (3.1)	20.8 (3.4)	0.001
Weight gain during pregnancy (kg)	11.2 (5.0)	11.1 (5.0)	10.8 (4.9)	<0.001
Multipara (%)	1 (0.8)	411 (10.6)	8,004 (29.8)	<0.001
Pre-existing hypertension (%)	0 (0.0)	3 (0.1)	65 (0.2)	0.110
Pre-existing diabetes or gestational diabetes (%)	0 (0.0)	41 (1.1)	407 (1.5)	0.035
Smoking				<0.001
Yes	15 (12.3)	465 (12.0)	2,162 (8.1)	
No	82 (67.2)	2,196 (56.9)	16,627 (61.9)	
Unanswered	25 (20.5)	1,202 (31.1)	8,057 (30.0)	
**Birth outcomes**				
Birthweight (g)	2891 (584)	2838 (577)	2856 (561)	0.152
Gestational age at birth (weeks)	38.3 (2.7)	38.3 (2.8)	38.3 (2.6)	0.792
Infant sex male (%)	70 (57.4)	2,006 (51.9)	13,700 (51.0)	0.226
**Pregnancy complications**				
Cesarean section (%)	21 (17.2)	630 (16.3)	5,549 (20.7)	<0.001
Unplanned cesarean section (%)	18 (14.8)	444 (11.5)	3,228 (12.0)	0.406
Preeclampsia (%)	4 (3.3)	99 (2.6)	705 (2.6)	0.878
Severe preeclampsia (%)	1 (0.8)	45 (1.2)	296 (1.1)	0.899
Preterm birth (%)	18 (14.8)	545 (14.1)	3,487 (13.0)	0.136
Very preterm birth (%)	4 (3.3)	147 (3.8)	865 (3.2)	0.165
Extremely preterm birth (%)	2 (1.6)	59 (1.5)	317 (1.2)	0.172
Low birth weight	24 (19.7)	763 (19.8)	5,034 (18.8)	0.324
Low Apgar at 5 minute (%)	5 (4.1)	107 (2.8)	551 (2.1)	0.005
Low pH of umbilical cord artery (%)	3 (2.5)	38 (1.0)	203 (0.8)	0.038
Severe laceration^b^ (%)	3 (3.0)	49 (1.5)	307 (1.4)	0.326
Stillbirth/early neonatal death (%)	0 (0.0)	28 (0.7)	184 (0.7)	0.630

Junior adolescent: Women aged ≤16.

Senior adolescent: Women aged 16–19.

Young adult: Women aged 20–24.

^a^p value using chi-square test for categorical variables and one-way ANOVA test for continuous variables.

^b^Analysis for this cell is n = 24,631 (excluded women who received cesarean-section).

When adjusted for confounders (model 1), adolescent women had significantly higher risk of adverse neonatal outcomes including preterm birth [adjusted risk ratio (aRR) 1.17, 95% confidence interval (95% CI), 1.08–1.27)], very preterm birth (aRR 1.32, 95% CI, 1.11–1.56), extremely preterm birth (aRR 1.39, 95% CI, 1.06–1.83), low birthweight (aRR 1.08, 95% CI, 1.01–1.15), and low Apgar score (aRR 1.41, 95% CI, 1.15–1.73) compared to women aged 20–24 years (Table 2). When additionally adjusted for maternal height, the mediator of interest, the results appeared slightly attenuated (model 2).

**Table 2:** Risk of neonatal outcomes associated with maternal age categories (versus aged 20–24 years).

Outcome	Maternal age	Women with complete data (n = 30,831)		
		Crude RR	Multivariate model 1^a^	Multivariate model 2^b^
Preterm birth (<37weeks)				
All adolescents		1.09 (1.00–1.18)	1.17 (1.08–1.27)	1.16 (1.07–1.26)
Junior adolescent		1.14 (0.74–1.74)	1.22 (0.81–1.82)	1.18 (0.79–1.76)
Senior adolescent		1.09 (1.00–1.18)	1.17 (1.08–1.27)	1.15 (1.06–1.25)
Very preterm birth (<32weeks)				
All adolescents		1.18 (0.99–1.39)	1.32 (1.11–1.56)	1.31 (1.10–1.55)
Junior adolescent		1.02 (0.39–2.67)	1.12 (0.45–2.80)	1.08 (0.43–2.72)
Senior adolescent		1.18 (1.00–1.40)	1.33 (1.12–1.57)	1.31 (1.10–1.55)
Extremely preterm birth (<28weeks)				
All adolescents		1.30 (0.99–1.70)	1.39 (1.06–1.83)	1.39 (1.06–1.83)
Junior adolescent		1.39 (0.35–5.51)	1.46 (0.37–5.71)	1.42 (0.36–5.55)
Senior adolescent		1.29 (0.98–1.70)	1.39 (1.05–1.84)	1.38 (1.04–1.82)
Small for gestational age				
All adolescents		0.92 (0.80–1.07)	0.92 (0.79–1.07)	0.88 (0.76–1.03)
Junior adolescent		0.96 (0.44–2.09)	0.90 (0.42–1.94)	0.82 (0.38–1.77)
Senior adolescent		0.92 (0.79–1.07)	0.92 (0.79–1.07)	0.89 (0.76–1.03)
Low birth weight				
All adolescents		1.05 (0.98–1.13)	1.08 (1.01–1.15)	1.05 (0.98–1.12)
Junior adolescent		1.05 (0.73–1.50)	1.03 (0.74–1.44)	0.97 (0.69–1.35)
Senior adolescent		1.05 (0.98–1.13)	1.08 (1.01–1.15)	1.05 (0.98–1.12)
Low Apgar (5 min, <7)				
All adolescents		1.37 (1.12–1.67)	1.41 (1.15–1.73)	1.41 (1.16–1.73)
Junior adolescent		2.00 (0.84–4.73)	2.06 (0.87–4.89)	2.03 (0.85–4.84)
Senior adolescent		1.35 (1.10–1.66)	1.39 (1.13–1.71)	1.39 (1.13–1.71)
IUFD/Early neonatal death				
All adolescents		1.03 (0.69–1.52)	1.06 (0.71–1.59)	1.07 (0.71–1.60)
Junior adolescent		none	—	—
Senior adolescent		1.06 (0.71–1.57)	1.10 (0.73–1.64)	1.09 (0.73–1.64)

Junior adolescent: Women aged ≤15.

Senior adolescent: Women aged 16–19.

All adolescent: Women aged ≤19.

^a^Adjusted by parity, pre-pregnancy BMI, gestational weight gain, maternal smoking, pre-existing hypertension, pre-existing diabetes or gestational diabetes, and year of delivery.

^b^Adjusted by parity, pre-pregnancy BMI, gestational weight gain, maternal smoking, preexisting hypertension, preexisting diabetes or gestational diabetes, year of delivery, and maternal height.

In the evaluation of maternal outcomes, adolescent women had significantly decreased risk of cesarean section (aRR 0.84, 95% CI, 0.78–0.91) as well as unplanned cesarean section (aRR 0.92, 95% CI, 0.84–1.01) compared to women aged 20–24 years (Table 3). After adjusting for maternal height, the estimated effect appeared slightly stronger in the protective direction.

**Table 3:** Risk of maternal outcomes associated with maternal age categories (versus aged 20–24 years).

Outcome	Maternal age	Women with complete data		
		Crude RR	Multivariate model 1^a^	Multivariate model 2^b^
Cesarean section^c^				
All adolescents		0.79 (0.73–0.85)	0.84 (0.78–0.91)	0.83 (0.77–0.89)
Junior adolescent		0.83 (0.56–1.23)	0.94 (0.64–1.38)	0.90 (0.62–1.33)
Senior adolescent		0.79 (0.73–0.85)	0.84 (0.78–0.90)	0.82 (0.76–0.89)
Unplanned cesarean section^c^				
All adolescents		0.96 (0.88–1.06)	0.92 (0.84–1.01)	0.90 (0.82–0.99)
Junior adolescent		1.23 (0.80–1.88)	1.19 (0.78–1.81)	1.13 (0.74–1.72)
Senior adolescent		0.96 (0.87–1.05)	0.91 (0.83–1.00)	0.89 (0.81–0.98)
Preeclampsia^c^				
All adolescents		0.98 (0.80–1.21)	0.87 (0.70–1.06)	0.84 (0.69–1.04)
Junior adolescent		1.25 (0.47–3.28)	1.11 (0.42–2.94)	1.07 (0.40–2.83)
Senior adolescent		0.98 (0.79–1.20)	0.86 (0.67–1.06)	0.84 (0.68–1.04)
Severe-preeclampsia^c^				
All adolescents		1.05 (0.77–1.43)	0.93 (0.68–1.27)	0.91 (0.67–1.24)
Junior adolescent		0.74 (0.11–5.25)	0.67 (0.09–4.73)	0.65 (0.09–4.62)
Senior adolescent		1.06 (0.77–1.44)	0.94 (0.69–1.29)	0.93 (0.68–1.27)
Severe-laceration^d^				
All adolescents		1.08 (0.81–1.45)	0.97 (0.73–1.31)	0.90 (0.67–1.21)
Junior adolescent		2.06 (0.67–6.32)	1.64 (0.53–5.05)	1.50 (0.50–4.52)
Senior adolescent		1.05 (0.78–1.42)	0.95 (0.70–1.29)	0.91 (0.67–1.23)

Junior adolescent: Women aged ≤15.

Senior adolescent: Women aged 16–19.

All adolescent: Women aged ≤19.

^a^Adjusted by parity, pre-pregnancy BMI, gestational weight gain, maternal smoking, pre-existing hypertension, pre-existing diabetes or gestational diabetes, and year of delivery.

^b^Adjusted by parity, pre-pregnancy BMI, gestational weight gain, maternal smoking, pre-existing hypertension, pre-existing diabetes or gestational diabetes, year of delivery, and maternal height.

^c^Analysis was conducted based on 30,831 women.

^d^Analysis was conducted based on 24,631 women who had a vaginal delivery.

We did not observe significant differences in effect on birth outcomes between junior and senior adolescent age groups (p-value > 0.05 for all outcomes), although the estimated risks of junior adolescent were larger for several outcomes such as low Apgar score and severe laceration. For all analyses, estimated effect of maternal age, as well as its change due to sequential adjusting, were similar between the main analyses results and sensitivity analyses using the dataset with imputed data (Appendix Tables 1, 2). Small, but significant, differences in maternal characteristics (i.e. smoking, pre-pregnancy BMI), birth outcomes (i.e. birthweight, gestational age at birth), and proportion of women with pregnancy complications (i.e. preterm birth, low birth weight, cesarean section) were observed between women who had missing data and those who did not (Appendix Table 3).

Mediation analysis was conducted for the following outcomes that showed significant or suggestive associations with adolescent pregnancy: preterm birth, very preterm birth, extremely preterm birth, low birthweight, and low Apgar score. The total effect of adolescent pregnancy on these outcomes estimated through these models, which also take into account possible exposure-mediator interaction, were similar to those observed in the models which did not take into account such interaction. The mediating effect of maternal height on the association between adolescent pregnancy and risk of adverse birth outcomes were 10.5%, 4.3%, 1.9%, 45.5%, and 6.6% for preterm birth, very preterm birth, extremely preterm birth, low birthweight, and low Apgar score at 5 minutes, respectively (Table 4).

**Table 4:** Direct effects and indirect (mediated by maternal height) effects of adolescent pregnancy on adverse outcomes.

	Risk ratio (95% confidence interval)		
	All adolescents	Junior adolescent	Senior adolescent
Preterm birth			
Total effect aRR (95% CI)	1.17 (1.07–1.28)	1.35 (1.24–1.48)	1.16 (1.06–1.26)
Natural direct effect aRR (95% CI)	1.15 (1.05–1.26)	1.30 (1.19–1.42)	1.14 (1.05–1.25)
Natural indirect effect aRR (95% CI)	1.02 (1.00–1.03)	1.04 (1.01–1.07)	1.02 (1.00–1.03)
% Mediated by height	10.5	10.6	10.0
Very preterm birth			
Total effect aRR (95% CI)	1.32 (1.11–1.57)	1.65 (1.40–1.95)	1.29 (1.09–1.52)
Natural direct effect aRR (95% CI)	1.30 (1.06–1.62)	1.62 (1.37–1.92)	1.27 (1.07–1.51)
Natural indirect effect aRR (95% CI)	1.01 (0.98–1.04)	1.02 (0.96–1.08)	1.01 (0.98–1.04)
% Mediated by height	4.3	2.9	4.3
Extremely preterm birth			
Total effect aRR (95% CI)	1.39 (1.05–1.84)	1.84 (1.41–2.40)	1.36 (1.05–1.77)
Natural direct effect aRR (95% CI)	1.38 (1.04–1.84)	1.83 (1.40–2.39)	1.35 (1.03–1.77)
Natural indirect effect aRR (95% CI)	1.01 (0.96–1.06)	1.01 (0.92–1.10)	1.01 (0.97–1.05)
% Mediated by height	1.9	1.0	2.3
Low birth weight			
Total effect aRR (95% CI)	1.08 (1.00–1.64)	1.16 (1.08–1.25)	1.07 (1.00–1.15)
Natural direct effect aRR (95% CI)	1.04 (0.96–1.12)	1.07 (1.00–1.15)	1.04 (0.96–1.11)
Natural indirect effect aRR (95% CI)	1.04 (1.02–1.05)	1.08 (1.05–1.11)	1.03 (1.02–1.05)
% Mediated by height	45.5	50.9	48.3
Low Apgar score at 5 min			
Total effect aRR (95% CI)	1.41 (1.15–1.74)	2.05 (1.68–2.50)	1.39 (1.15–1.69)
Natural direct effect aRR (95% CI)	1.38 (1.12–1.70)	1.89 (1.55–2.29)	1.36 (1.12–1.66)
Natural indirect effect aRR (95% CI)	1.03 (0.99–1.06)	1.09 (1.02–1.16)	1.02 (0.99–1.05)
% Mediated by height	6.6	8.3	5.6

Junior adolescent: Women aged ≤15.

Senior adolescent: Women aged 16–19.

All adolescent: Women aged ≤19.

aRR: adjusted risk ratio.

All analyses adjusted for parity, pre-pregnancy BMI, gestational weight gain, maternal smoking, pre-existing hypertension, pre-existing diabetes or gestational diabetes, and year of delivery.

## Discussion

In our study, we found that adolescent women have increased risks for neonatal adverse outcomes such as preterm birth, low birthweight, and low Apgar score, while showing a decreased risk of cesarean section and no association with other maternal adverse outcomes such as preeclampsia and severe laceration. We also found that the increased risk of adverse neonatal outcomes among adolescent women was partially mediated by shorter maternal height, suggesting a role for maternal physical immaturity. This is the first epidemiological study to evaluate the contribution of maternal physical immaturity on the association between adolescent pregnancy and adverse birth outcomes, as well as the first to show adverse birth outcomes among Japanese adolescent women.

In line with previous studies^10–13,15^, we observed increased risks of preterm delivery, low birthweight and low Apgar score^10^ associated with adolescence compared to women of 20–24 years of age. While previous studies speculated this association to be due to both physical and social factors, analysis to estimate their contributions had not been conducted. In our study, we used mediation analysis^30^ to assess the mediating effect of height in this association. We focused on maternal height as a mediating factor, as adolescent women are shorter compared to older women, and shorter women have been reported to have an increased risk of preterm birth and low birthweight^19,20^, which is suggested to be due to smaller pelvic size.

In our study, we observed that maternal height significantly mediated the association between adolescent pregnancy and preterm birth, low birthweight and low Apgar score. These results support a hypothesis that shorter height, which relates to smaller pelvic size among adolescent women^32^, is partly responsible for the observed increased risk of preterm birth; preterm infants are also at a higher risk of being born low birthweight as well as with neonatal asphyxia. We also observed that the mediated effect was smaller for very preterm birth (4.3%) and extremely preterm birth (1.9%) compared to total preterm birth (10.5%). As limited pelvic size would only be a problem in late pregnancy when the fetus is quite large, these observations are also consistent with the interpretation that physical immaturity contributes to those adverse effects of pregnancy in adolescents.

It is also noteworthy that while the observed mediated effect was significant, the estimated effect size was quite small for all outcomes (aRR 1.01 to 1.04), with the unexplained direct effect much larger (aRR 1.04 to 1.38). These findings suggest that the majority of the adverse effect observed in adolescent pregnancies may not be due to the physical constraints of an adolescent body, but may rather be driven by socially-derived risk factors, such as inadequate prenatal or delivery care^33^ which has been repeatedly observed among pregnancies of adolescent women^34^.

Consistent with previous studies^10–13,15^, we found decreased risk of cesarean section (including unplanned cesarean section) associated with adolescent pregnancies. However, the explanation for this observed decrease in risk is not yet clear. A previous report showed an increased risk for cesarean section in adolescent women when restricted to those with indication for presumed cephalo-pelvic disproportion, suggesting that differences in indication for cesarean section may be confounding the results^4^. Another study showed increased risk of failure to progress or cephalop-pelvic disproportion in adolescent women^11^. Thus, we cannot rule out the possibility that differences in indication for cesarean section confounded our findings as we did not have appropriate data for a thorough evaluation. Further assessment of this issue is warranted.

When comparing risk of adverse birth outcomes between junior and senior adolescent women, we did not detect significant differences between the two groups, although the estimated risk was larger among junior adolescents for several of the outcomes such as preterm birth and severe laceration. As larger studies have reported junior adolescents have greater risk of preeclampsia, preterm birth, low birthweight, and neonatal asphixia^10,24,25^, the inability of our study to detect a clear pattern may have been due to limited statistical power in the analysis of adolescents under 15 years of age.

The strengths of our study include the implementation of mediation analysis to clarify the role of physical factors in the association between adolescent pregnancy and adverse birth outcomes, as well as the utilization of a large nation-wide clinical database in Japan which enabled us to conduct complex analyses on adolescent pregnancies. Nonetheless, the current study has some limitations. First, we did not have maternal age at conception and used maternal age as delivery as its proxy. However, we believe its influence on our results would be relatively small considering the pregnancy period is 7–10 months. Second, our analyses were conducted using a database based on Japanese tertiary hospitals, which may have resulted in the inclusion of more women with higher risk pregnancies compared with the general population. However, we excluded women with pre-existing clinical risk factors of adverse birth outcome, such as maternal diabetic disorder or pre-existing hypertension, multiple pregnancy, and fetal congenital anomaly, to maximize generalizability of findings. In addition, termination of pregnancies^5^, which adolescent pregnancies are also at higher risk, were not captured in our database, thus our analysis based on pregnancies past 22 weeks may be underestimating the true adverse risk associated with adolescent pregnancies. Future prospective population based studies are encouraged to confirm the reproducibility of our findings. Third, we relied on maternal height as a proxy for physical immaturity in this study. However, adolescent women may have further biological constraints due to immaturity (e.g. endocrinological immaturity, uterine immaturity) which are not captured through height. Studies investigating the extent to which other constraints may account for the adverse effect of adolescent pregnancy on birth outcomes are needed. Fourth, while our study indirectly suggests that social and lifestyle factors also contribute a significant proportion of the effect of adolescent pregnancy on adverse birth outcomes (as the percent mediation levels through maternal height is modest) this effect may have been underestimated due to residual confounding. We were unable to account for socio-economic status, lifestyle factors including undernutrition leading to lower maternal height, as well as adverse birth outcomes^33,34^ due to of the lack of information in our database. Furthermore, we were able to adjust for smoking status only crudely using a binary categorization (smoked during pregnancy: yes/no) despite previous studies indicating that frequency and duration may influence on birth outcomes. Future studies are need to evaluate whether social factors and detailed smoking status may be responsible for the remaining unexplained increase in risk. Fifth, as the main interest of this study focused on the role of maternal height in explaining the adverse effects of adolescent pregnancy, we did not conduct detailed analysis of how the effect of adolescent pregnancy may vary by maternal characteristics and assumed the effects were similar. Observing that previous studies has reported that the adverse effects of adolescent pregnancy was greater among multiparous women compared to primiparous women^35,36^, this assumption may have be violated in our study. Future studies focusing on effect modification by maternal characteristics are encouraged.

In conclusion, we found an increased risk of preterm birth, low birthweight, and low Apgar score associated with adolescent pregnancies in Japanese women. We provide evidence that while physical immaturity (measured by maternal height) does significantly contribute to this association as a mediator, the modest levels of estimated mediation leaves room for a potentially larger role for social and lifestyle-related factors that are associated with adolescence.

## Supplementary information

supplemental Table 1–3

## Data Availability

The datasets generated during and/or analyzed during the current study are available from the corresponding author on reasonable request.
