# Supplementary material for: Association between adolescent pregnancy and adverse birth outcomes, a multicenter cross sectional Japanese study
Source: Sci Rep. 2019 Feb 20;9:2365. doi: 10.1038/s41598-019-38999-5 (PMC6382879; doi:10.1038/s41598-019-38999-5)
Supplement: Supplementary file 1 — supplemental Table 1–3 [file 41598_2019_38999_MOESM1_ESM.docx]

Supplementary Information for Ogawa K, Matsushima S, Urayama K, Kikuchi N, Nakamura N, Tanigaki S, Sago H, Satoh S, Saito S, Morisaki N. Association between adolescent pregnancy and adverse birth outcomes, a multicenter cross sectional Japanese study:

Appendix Table 1-3

| **Appendix table 1. Risk of neonatal outcomes associated with maternal age categories using imputed data (reference group: age 20-24 years)** | | | | |
| --- | --- | --- | --- | --- |
| Outcome | Maternal age | Risk ratio (95% confidence interval) | | |
|  |  | Crude | Multivariate model 1^a^ | Multivariate model 2^b^ |
|  |  |  |  |  |
| Preterm birth (<37weeks) | |  |  |  |
|  | All adolescents | 1.11 (1.05-1.18) | 1.17 (1.10-1.24) | 1.16 (1.09-1.23) |
|  | Junior adolescent | 1.38 (1.05-1.82) | 1.45 (1.11-1.89) | 1.41 (1.08-1.84) |
|  | Senior adolescent | 1.11 (1.04-1.17) | 1.16 (1.09-1.24) | 1.15 (1.08-1.22) |
| Very preterm birth (<32weeks) | |  |  |  |
|  | All adolescents | 1.26 (1.13-1.42) | 1.38 (1.23-1.55) | 1.37 (1.21-1.54) |
|  | Junior adolescent | 1.27 (0.69-2.34) | 1.39 (0.77-2.49) | 1.35 (0.75-2.43) |
|  | Senior adolescent | 1.26 (1.12-1.42) | 1.38 (1.22-1.56) | 1.37 (1.21-1.54) |
| Extremely preterm birth (<28weeks) | | |  |  |
|  | All adolescents | 1.41 (1.17-1.69) | 1.51 (1.25-1.83) | 1.50 (1.24-1.81) |
|  | Junior adolescent | 1.36 (0.51-3.61) | 1.43 (0.55-3.76) | 1.39 (0.53-3.66) |
|  | Senior adolescent | 1.41 (1.17-1.70) | 1.52 (1.25-1.84) | 1.50 (1.24-1.82) |
| Small for gestational age | |  |  |  |
|  | All adolescents | 0.97 (0.86-1.08) | 0.93 (0.83-1.05) | 0.90 (0.81-1.01) |
|  | Junior adolescent | 0.67 (0.32-1.39) | 0.63 (0.30-1.29) | 0.58 (0.28-1.19) |
|  | Senior adolescent | 0.97 (0.87-1.09) | 0.94 (0.84-1.06) | 0.91 (0.81-1.03) |
| Low birth weight | |  |  |  |
|  | All adolescents | 1.08 (1.02-1.13) | 1.07 (1.02-1.13) | 1.05 (0.99-1.10) |
|  | Junior adolescent | 1.07 (0.81-1.42) | 1.04 (0.79-1.36) | 0.98 (0.75-1.28) |
|  | Senior adolescent | 1.08 (1.02-1.14) | 1.08 (1.02-1.13) | 1.05 (1.00-1.11) |
| Low Apgar (5 min, <7) | |  |  |  |
|  | All adolescents | 1.25 (1.09-1.45) | 1.30 (1.13-1.51) | 1.29 (1.12-1.49) |
|  | Junior adolescent | 1.70 (0.90-3.24) | 1.76 (0.93-3.35) | 1.73 (0.91-.29) |
|  | Senior adolescent | 1.24 (1.07-1.43) | 1.29 (1.11-1.49) | 1.28 (1.10-1.48) |
| IUFD/Early neonatal death | |  |  |  |
|  | All adolescents | 1.19 (0.93-1.53) | 1.22 (0.95-1.57) | 1.22 (0.95-1.57) |
|  | Junior adolescent | 0.54 (0.08-3.82) | 0.54 (0.08-3.74) | 0.54 (0.08-3.76) |
|  | Senior adolescent | 1.22 (0.95-1.56) | 1.24 (0.96-1.59) | 1.24 (0.96-1.60) |
| Analysis was conducted based on 53,301 women | | | | |
| Multiple imputation used to impute missing values of maternal height(n=17,449), pre-pregnancy BMI (n=19,387) , and smoking status (n=29,034), (100 iterations) | | | | |
| Junior adolescent: Women aged ≤15 | | |  |  |
| Senior adolescent: Women aged 16-19 | | |  |  |
| All adolescent: Women aged ≤19 | | |  |  |
| a: Adjusted by parity, pre-pregnancy BMI, gestational weight gain, maternal smoking, pre-existing hypertension, pre-existing diabetes or gestational diabetes, and year of delivery | | | | |
| b: Adjusted by parity, pre-pregnancy BMI, gestational weight gain, maternal smoking, pre-existing hypertension, pre-existing diabetes or gestational diabetes, year of delivery, and maternal height | | | | |

| **Appendix table 2. Risk of maternal outcomes associated with maternal age categories using imputed data (reference group: age 20-24 years)** | | | | |
| --- | --- | --- | --- | --- |
| Outcome | Maternal age | Risk ratio (95% confidence interval) | | |
|  |  | Crude | Multivariate model 1^a^ | Multivariate model 2^b^ |
|  |  |  |  |  |
| Cesarean section ^c^ | |  |  |  |
|  | All adolescents | 0.82 (0.77-0.86) | 0.86 (0.81-0.90) | 0.84 (0.80-0.89) |
|  | Junior adolescent | 0.80 (0.59-1.08) | 0.87 (0.65-1.17) | 0.84 (0.63-1.14) |
|  | Senior adolescent | 0.82 (0.77-0.86) | 0.86 (0.81-0.90) | 0.84 (0.80-0.89) |
| Unplanned cesarean section ^c^ | |  |  |  |
|  | All adolescents | 0.96 (0.90-1.03) | 0.92 (0.86-0.98) | 0.90 (0.84-0.96) |
|  | Junior adolescent | 1.05 (0.74-1.48) | 0.98 (0.70-1.39) | 0.94 (0.67-1.33) |
|  | Senior adolescent | 0.96 (0.90-1.03) | 0.91 (0.85-0.98) | 0.90 (0.84-0.96) |
| Preeclampsia ^c^ | |  |  |  |
|  | All adolescents | 1.05 (0.91-1.22) | 0.92 (0.79-1.06) | 0.90 (0.78-1.05) |
|  | Junior adolescent | 1.03 (0.47-2.27) | 0.89 (0.40-1.96) | 0.86 (0.39-1.90) |
|  | Senior adolescent | 1.05 (0.91-1.22) | 0.92 (0.79-1.07) | 0.91 (0.78-1.05) |
| Severe-preeclampsia ^c^ | |  |  |  |
|  | All adolescents | 1.07 (0.85-1.34) | 0.95 (0.75-1.20) | 0.94 (0.74-1.19) |
|  | Junior adolescent | 0.43 (0.06-3.03) | 0.38 (0.05-2.71) | 0.37 (0.05-2.64) |
|  | Senior adolescent | 1.09 (0.86-1.37) | 0.97 (0.77-1.23) | 0.96 (0.76-1.21) |
| Severe-laceration ^d^ | |  |  |  |
|  | All adolescents | 1.17 (0.94-1.45) | 1.02 (0.82-1.27) | 0.99 (0.79-1.23) |
|  | Junior adolescent | 2.82 (1.36-5.85) | 2.33 (1.12-4.82) | 2.19 (1.06-4.51) |
|  | Senior adolescent | 1.11 (0.89-1.39) | 0.97 (0.78-1.22) | 0.95 (0.75-1.19) |
| Multiple imputation used to impute missing values of maternal height(n=17,449), pre-pregnancy BMI (n=19,387) , and smoking status (n=29,034), (100 iterations) | | | | |
| Junior adolescent: Women aged ≤15 | | |  |  |
| Senior adolescent: Women aged 16-19 | | |  |  |
| All adolescent: Women aged ≤19 | | |  |  |
| a: Adjusted by parity, pre-pregnancy BMI, gestational weight gain, maternal smoking, pre-existing hypertension, pre-existing diabetes or gestational diabetes, and year of delivery | | | | |
| b: Adjusted by parity, pre-pregnancy BMI, gestational weight gain, maternal smoking, pre-existing hypertension, pre-existing diabetes or gestational diabetes, year of delivery, and maternal height | | | | |
| c: Analysis was conducted based on 53,301 women | | | | |
| d: Analysis was conducted based on 42,365 women who experienced vaginal delivery | | | | |

| **Appendix table 3. Maternal and infant characteristics among women with complete data and women with imcomplete data.** | | | | | | |
| --- | --- | --- | --- | --- | --- | --- |
|  |  |  |  |  |  |  |
| Mean (SD) or n (%) | | | **The number of available data among women with imcomplete data** | **Women with conplete data (n=30,831^a^)** | **Women with imconplete data** | **p^b^** |
|  |  |  |  |  |  |  |
| **Maternal characteristics** | | |  |  |  |  |
|  | Maternal age (years) | | 22,470 | 21.9 (2.0) | 21.9 (2.0) | <0.001 |
|  | Maternal weight (kg) | | 4,930 | 51.4 (8.9) | 52.5 (11.1) | <0.001 |
|  | Maternal height (cm) | | 5,021 | 157.3 (5.5) | 157.5 (5.7) | 0.012 |
|  | Weight gain during pregnancy (kg) | | 1,764 | 10.8 (4.9) | 10.6 (5.6) | 0.036 |
|  | BMI (kg/m^2^) | | 3,083 | 20.7 (3.3) | 21.4 (4.4) | <0.001 |
|  |  |  |  |  |  |  |
|  | Multipara (%) | | 14,525 | 8,416 (27.3) | 6,109 (27.3) | 0.969 |
|  | Pre-existing hypertension (%) | | 22,470 | 68 (0.2) | 45 (0.2) | 0.615 |
|  | Pre-existing diabetes or gestational diabetes (%) | | 22,470 | 448 (1.5) | 238 (1.1) | <0.001 |
|  | Smoking (%) | | 22,470 |  |  | <0.001 |
|  |  | Yes |  | 2,642 (8.6) | 1,214 (5.4) |  |
|  |  | No |  | 18,905 (61.3) | 14,983 (66.7) |  |
|  |  | Unanswered |  | 9,284 (30.1) | 6,273 (27.9) |  |
|  |  |  |  |  |  |  |
| **Birth outcomes** | | |  |  |  |  |
|  | Birthweight (g) | | 22,470 | 2854 (63) | 2811 (611) | <0.001 |
|  | Gestational age at birth (weeks) | | 22,470 | 38.3 (2.6) | 38.1 (2.9) | <0.001 |
|  | Infant sex male (%) | | 22,470 | 15,776 (51.2) | 11,703 (52.1) | 0.037 |
|  |  |  |  |  |  |  |
| **Pregnancy complications** | | |  |  |  |  |
|  | Cesarean section (%) | | 22,470 | 6,200 (20.1) | 4,736 (21.1) | 0.006 |
|  | Unplanned cesarean section (%) | | 22,470 | 3,690 (12.0) | 2,931 (13.0) | <0.001 |
|  | Preeclampsia (%) | | 22,470 | 808 (2.6) | 654 (2.9) | 0.043 |
|  | Severe preeclampsia (%) | | 22,470 | 342 (1.1) | 245 (1.1) | 0.836 |
|  | Preterm birth (%) | | 22,470 | 4,050 (13.1) | 3,495 (15.6) | <0.001 |
|  | Very preterm birth (%) | | 22,470 | 1,016 (3.3) | 1,008 (4.5) | <0.001 |
|  | Extremely preterm birth (%) | | 22,470 | 378 (1.2) | 392 (1.7) | <0.001 |
|  | Low birth weight (%) | | 22,470 | 5,821 (18.9) | 3,586 (16.0) | <0.001 |
|  | Low Apgar at 5 minute (%) | | 22,470 | 663 (2.2) | 697 (3.1) | <0.001 |
|  | Low pH of umbilical cord artery (%) | | 22,470 | 244 (0.8) | 154 (0.7) | 0.160 |
|  | Severe laceration (%) | | 17,734 | 359 (1.5) | 246 (1.4) | 0.547 |
|  | Stillirth/early neonatal death (%) | | 22,470 | 212 (0.7) | 262 (1.2) | <0.001 |
| a: Analysis was conducted based on 24,631 women who experienced vaginal delivery. | | | | |  |  |
| b: Using student t test for continuous variables and chi-squared test for categorical variables. | | | | | |  |
